# Remarks on the Work of the Buffalo Laboratory on the Investigation of Cancer1Read at the thirty-third annual meeting of the Medical Association of Central New York, held at Rochester, N. Y., October 16, 1900.

**Published:** 1901-03

**Authors:** Harvey R. Gaylord

**Affiliations:** Buffalo, N. Y.


// BUFFALO MEDICAL JOURNAL.

VOL. XL.—LVI. MARCH, 1901. No. 8.

### Original Communications.

### REMARKS ON THE WORK OF THE BUFFALO LABORATORY ON THE INVESTIGATION OF CANCER.[1]

By HARVEY R. GAYLORD, M. D., Buffalo, N. Y.

MY appearance before you today is rather unexpected and I fear that coming unprepared, it will be impossible for me to give you more than a general statement as to the present status of the numerous investigations which are being pursued to determine the nature of cancer, and some idea of the relation which our laboratory holds to the general subject.

There probably has never been a period of greater activity in this field and investigations are being pursued in all parts of the world. The publications which have appeared as a result of this activity have been so extensive that it is extremely difficult to keep pace with them. Beside the great mass of material which comes to our hands, we have encountered and still continue to encounter unusual difficulties in interpreting the work of men approaching the subject from various standpoints. For this reason it has been necessary in some cases for us to go directly to the investigator and personally inspect his material.

The present activity in this field of research is unquestionably due, first of all, to the general belief that the number of cancer cases is slowly but surely increasing. This belief is based upon statistics which have been collected over a period of time and which, in their present form, leave no reasonable doubt that there is an actual increase in the number of cancer cases in all parts of the civilised world.

1. Read at the thirty-third annual meeting of the Medical Association of Central New York, held at Rochester, N. Y., October 16, 1900.

Another motive which has served to stimulate scientific research has been the feeling among scientists that so far as cancer is concerned, the scientific profession possesses less satisfactory knowledge about this disease than any other. It is an extremely difficult problem and one upon which it is impossible to dogmatise or speculate.

Naturally, the most burning question in connection with this disease is whether it is of parasitic nature or not. There are some facts which seem to make it almost impossible that cancer can be the result of parasitic activity, and at the same time these facts may not be in reality more stubborn than many others which have been overcome in the solution of other diseases which we, today, know are of parasitic origin. In attempting to solve the nature of the disease and especially to determine whether it is of parasitic nature, the attack has been principally along two lines, and has led, at least at the present time, to two opposing factions among those who advocate the parasitic theory. One group of investigators believes that the parasite of cancer will have to be found in the vegetable kingdom; while the other believes that in all probability certain bodies and formations which are found in cancer are animal parasites. A few years ago the literature contained many articles describing bodies and forms in cancer, which the authors attempted to interpret as protozoa. The idea that the parasite of cancer was a protozoan has fallen, during the last year or two, more into the background, owing to the activity of certain Italian investigators, who have attempted to show that these bodies are not of animal, but of vegetable nature and that the parasite of cancer belongs in the vegetable kingdom. Within the last year the most interesting publications have been by those who again advocate the animal nature of the parasite.

It will be seen that under these conditions, a well equipped institution with no axe to grind, and supported by the state, is extremely necessary and can serve the purpose, in a certain sense, of a clearing house for all of these conflicting investigations. This function we have attempted to perform and thus far, although we have carried out much original research, we have busied ourselves principally in the consideration of the relation of certain vegetable organisms to cancer. A report covering these investigations will be made to the legislature during the coming session and will later be published more in full.

Certain conditions are absolutely essential to the success of such a laboratory as the one in Buffalo. First of all, time is probably the greatest element of success. Cancer is a disease of great chronicity and the results which we have thus far been able to obtain in animals, which have thrown any light upon the question, have, as a rule, only been obtained after a considerable period of time. For this reason it is absolutely necessary that our work should be continued for a period of years if any profitable results are to be obtained. It requires a large and properly manipulated material, sufficient equipment and sufficient quarters; this last I am fortunately in a position to state will be provided for us during the coming year through the philanthropy of private individuals in Buffalo.

It may be of interest to you to know that the foundation of this laboratory has created widespread interest even in foreign lands, and since the period of its foundation Germany has formed a national cancer society, which proposes to undertake the work of collecting reliable statistics upon this subject. The English have, likewise, established a national cancer society and two institutions in London have provided funds for research. A benevolent lady of Boston has bequeathed to Harvard University a sum of $200,000, the income of which is to be devoted to the investigation of this subject. A year ago when in Europe, I found that all scientists were keenly interested in the innovation, so far as America is concerned, of a state undertaking work of this nature and I can assure you it has reflected no little credit on the State of New York, that she has been foremost in appropriating money for abstract investigation of scientific subjects of this nature.

I feel that my remarks have been very rambling, but what I wish most of all to impress upon this society, is that we require the support of the profession of the state, and that this support should be exerted upon the legislators who will pass upon the future appropriations for the continuation of the work. It, therefore, rests with the profession of the state to see that institutions of this sort are supported and continued, and it is especially necessary that all physicians should take an active interest, as we have been informed that there is a tendency among the legislators to raise the question at the next meeting of the legislature of the propriety of the state supporting scientific research at all. It is unnecessary for me to point out to you that although such a stand would be injurious to the interests of the people, the results would be especially unfortunate for the members of the medical profession. Certainly a profession which serves the people as does ours has a right to expect something from the state. For this reason I urge upon this society the necessity for support, if our work is to be continued. It requires years in a laboratory of this sort before definite conclusions can be reached; a fact, which legislators do not always appreciate. It would be unfortunate if the State of New York, having once taken a stand as advanced as that involved in establishing a laboratory for special research, should abandon the work, and I am sure that if the matter is intelligently presented to the legislators that they will appreciate not only the necessity, but the desirability of maintaining an institution of this character.

23 Irving Place.

DISCUSSION.

Dr. J. W. Whitbeck, Monroe—We have heard the very interesting remarks of Dr. Gaylord on the progress of this work, and I need hardly urge upon this association the importance of aiding in this great movement. You all know the feeling of the people at large in regard to this matter, based mainly upon the expense incurred in carrying on such work, and it is in response to this feeling of the mass of the people that the legislators are influenced, as it seems to us, to not promote this work any more rapidly than the public demand it. It is unnecessary for me to say that it is proper for this association to take an active stand in this matter, in order to promote this great work, and by the appointment of a committee, whose duty it shall be to address the different members of this Association from time to time, and so keep them alive to the interests of this matter, that they may reach their representatives in the legislature. By having such a committee to watch opposing legislation and also to promote favorable legislation we, I think, will do great good in this matter, not only as physicians, not only as a scientific body, but also as patriots. I would therefore, move you, Mr. Chairman, that the chair appoint a committee of five, it being distinctly understood that Dr. Gerin shall be a member of this committee, whose duty it shall be to promote this great work.

Seconded by Dr. A. L. Benedict, Erie.

The President.—Are there any remarks upon the motion?

Dr. N. Jacobson, Onondaga—I would like to ask what the purpose of this committee shall be, whether it is understood that this committee is appointed for one specific purpose or for the general subject of state support for scientific investigation. It seems to me that we want to be a little broader than the question that is before us. While we all admit that there is no more serious condition that afflicts mankind than malignant disease, while we recognise that with all the research, common as it is with us every day, it still eludes us, and that there is no more important study here, still, those of us who are interested in scientific work that is to be done by the various institutions of the state, feel that the state support for this institution is not only justified for itself, but is opening the way for the other institutions to claim support, and the support, not that they crave, but that they deserve, the support that the state owes all educational institutions, to coöperate with them and assist them in every way towards the end. They have in view the education of the people, the education of the profession and the good of mankind; and therefore, I feel that the purpose of this committee should not be restricted in any way, but that it should be a broad one, one that shall clear away any obstruction that it can that is in their road in the way of legislation, which looks toward the coöperation with scientific institutions and the advancement of knowledge in our work.

Dr. WHITBECK—I intend to have it cover that ground, namely, that this committee should interest itself in all scientific work and promote it, as far as in its power. Carried.

The President appointed as such committee, John W. Whitbeck, E. V. Stoddard, Monroe; Nathan Jacobson, Onondaga; John Gerin, Cayuga; C. M. Lee, Oswego.

